# Effects of Melatonin Supplementation on Sleep Quality in Breast Cancer Patients: A Systematic Review and Meta-Analysis

**DOI:** 10.3390/healthcare11050675

**Published:** 2023-02-24

**Authors:** Kyoungsan Seo, Jin-Hee Kim, Dallong Han

**Affiliations:** 1 College of Nursing, Chungnam National University, Daejeon 35015, Republic of Korea; 2 Department of Biomedical Laboratory Science, Cheongju University, Cheongju 28503, Republic of Korea; 3 Department of Nursing, Cheongju University, Cheongju 28503, Republic of Korea

**Keywords:** breast cancer, melatonin, meta-analysis, sleep

## Abstract

Evidence on the effectiveness of melatonin in breast cancer patients suffering from sleep disturbances is contradictory, and there have been no meta-analyses on its use in humans with breast cancer. This study investigated the melatonin supplementation effectiveness in alleviating sleep disturbances in breast cancer patients. We searched Embase, PubMed, MEDLINE, CINAHL, Cochrane Library, Google Scholar, and Clinical trial.org databases for relevant reports by following PRISMA guidelines and collected clinical experimental studies of melatonin supplementation in breast cancer patients. Breast cancer for the population, melatonin supplementation for intervention, including sleep indicator, cancer treatment-related symptoms for outcomes, and clinical trial for humans were the searched keywords. Among the 1917 identified records, duplicates and irrelevant articles were excluded. Among the 48 full-text articles assessed, 10 studies met the criteria for inclusion in a systematic review, and five studies had sleep-related indicators and were included in the meta-analysis after quality assessment. The estimated average effect size (Hedges’ g) was −0.79 (*p <* 0.001) in a random-effects model, thus indicating that melatonin supplementation had a moderate effect in ameliorating sleep quality in breast cancer patients. Pooled data from studies on melatonin supplementation indicate that melatonin administration may alleviate sleep problems related to treatments in breast cancer patients.

## 1. Introduction

Patients diagnosed with hormone-related cancer often experience various symptoms that appear during and after treatment, such as the sequelae of surgery, chemotherapy, and hormone therapy, in addition to cancer symptoms [1,2,3,4,5]. Particularly, sleep disturbances in cancer patients may manifest from anxiety owing to the prognosis; however, in cancer patients receiving hormone therapy, sleep problems may be caused by drug-induced menopausal symptoms [6]. Additionally, sleep disorders or melatonin level imbalances are considered factors that cause hormone-related cancer [7].

Sleep disturbances affect the quality of life and treatment compliance of cancer patients [4]. Moreover, this presents an opportunity for patients to try various complementary and alternative medicine to resolve symptoms of sleep disorders [8,9]. Therefore, improving sleep quality is important because it may increase treatment adherence, improve the quality of life of patients, and prevent complications in hormone-related cancer patients.

Several treatments and strategies have been proposed to improve the sleep and quality of life of breast cancer patients, including melatonin supplementation. However, owing to their interaction with drugs used for breast cancer treatment, naturally derived melatonin supplementation is administered rather than hypnotics [10]. Melatonin (N-acetyl-5-methoxytryptamine) is a hormone that is synthesized and secreted by the pineal gland mainly at night [11]. Several studies have reported the effectiveness and safety of melatonin administration in subjects with primary sleep disorders [12,13]. However, results on the effectiveness of melatonin in breast cancer patients suffering from insomnia differ across studies and cancer type [14], and no meta-analysis has been conducted on its use in humans with breast cancer [13,15]. Thus, there is a need for research that can assess the evidence on the effectiveness of melatonin supplementation for the treatment of sleep disturbances in breast cancer patients.

In this study, we reviewed the context and adverse effects of melatonin intervention provided to breast cancer patients and investigated whether melatonin supplementation is effective in improving sleep quality in breast cancer patients via a systematic review and meta-analysis.

## 2. Materials and Methods

This systematic review was conducted in accordance with the PRISMA guidelines [16].

### 2.1. Research Questions

The research questions were as follows: “What are the characteristics of melatonin administration and subjects in each study?”; “were sleep difficulties in breast cancer patients significantly alleviated after melatonin administration compared with those in a control group?”; and “were there any adverse effects after the administration of melatonin?”.

### 2.2. Search Strategy

We searched Embase, PubMed, MEDLINE, CINAHL, Cochrane Library, Google Scholar, and clinical trial.org databases for relevant reports. We used MesH or EMTREE terms that were confirmed by a preliminary investigation. The search terms were as follows: {“breast neoplasms” [MeSH Terms] OR (“breast” [All Fields] AND “neoplasms” [All Fields]) OR “breast neoplasms” [All Fields] OR (“breast” [All Fields] AND “cancer” [All Fields]) OR “breast cancer” [All Fields] [OR] breast carcinoma [OR] breast tumor [OR] breast malignancy [OR] mammary carcinoma} AND {Melatonin [title/abstract] “melatonin” [MeSH Terms] OR “melatonin” [All Fields] OR “melatonin s” [All Fields] OR “melatonine” [All Fields] OR “melatonins” [All Fields]) AND (“supplemental” [All Fields] OR “supplementing” [All Fields] OR “supplementation” [All Fields] OR “supplementation s” [All Fields] OR “supplementations” [All Fields] OR “supplementation” [All Fields])} AND Filters: Humans OR [human]/lim.

We recognized all academic articles published from the commencement of the database to February 2022. Regarding the use of the clinical trial registration platform and Google Scholar, manual searching was performed to improve the sensitivity of the data search. We eliminated duplicate records after compiling all relevant data in one reference management program tool, EndNote X9. By screening the title and abstract of the remaining articles, irrelevant articles were excluded. Based on the selection criteria (see below), studies that could be included in the review and analysis were then assessed.

Three researchers worked independently to perform each procedure. Disagreements were settled by dialog and consensus. The clinical features of the individuals; administration of melatonin; comparison; outcome indicators; measurements; and key findings, including reported side effects, were retrieved during the qualitative synthesis. The number of samples and the outcomes by the group in each study were noted for the meta-analysis.

### 2.3. Selection Criteria

The PICO-SD questions served as the basis for including and excluding studies (population or participant, intervention, comparison, outcome, and study design). Studies that involved the administration of melatonin supplements and control groups met the selection criteria. Only human clinical trials where breast cancer had been identified were included. Pilot studies and preclinical experimental studies were excluded. After reading the entire article, studies that involved children or pregnant women were excluded. Only publications that investigated the sleep indicators of breast cancer patients were ultimately chosen for the meta-analysis among all available papers.

### 2.4. Risk-of-Bias Appraisal

The included studies were subjected to a risk-of-bias assessment to create a quantitative synthesis of the effects of sleep improvement. Risk-of-bias assessment was performed using the Cochrane risk-of-bias tool for randomized trials, “https://www.riskofbias.info/welcome/rob-2-0-tool/current-version-of-rob-2 (accessed on 29 June 2022) [17]”. The risk-of-bias was evaluated based on five criteria: the randomization process, deviations from the intended interventions, missing outcome data, measurement of the outcome, and selection of the reported result. Two evaluators worked independently to complete the evaluation after reading the original articles. The final appraisal findings were decided by discussions if there was a discrepancy in the evaluators’ scores.

### 2.5. Statistical Analysis

We employed Comprehensive Meta-Analysis (CMA) 3.0 software (Biostat, Englewood, NJ, USA) for the meta-analysis. It was assumed that each study was conducted with various participants in various regions, and the same amount and quality of intervention were not provided for each study. As a result of the analysis using the random effect model, we tried to generalize to breast cancer patients experiencing sleep problems. It was verified that it was statistically significant within the confidence interval using the Hedge’s g value from the results of sleep quality. The standardized effect size of changes was used when different scales were used [18]. Results were deemed statistically significant when *p* < 0.05 in a two-tailed test. The group’s pre-, post-value, difference value, or subjects’ number were extracted for each study. The values in various ways were integrated and analyzed through the CMA program at once. The results were displayed in the order of forest plot and effect size and visually confirmed. When the effect size was expressed as a negative value, it means that the quality of sleep is improved through intervention. RCT and non-RCT designs were analyzed together. Researchers read and judged the selection of papulations and the intervention contents in each publication. Additionally, I^2^ > 75%, 25% < I^2^ < 75% and I^2^ < 25% were determined to have high heterogeneity, moderate heterogeneity, and low heterogeneity, respectively [17]. For sensitivity analysis, the research design type was set as a subgroup, and the result value except for the study with a skewed value, was analyzed.

## 3. Results

### 3.1. Search Outcomes

**Figure 1:**
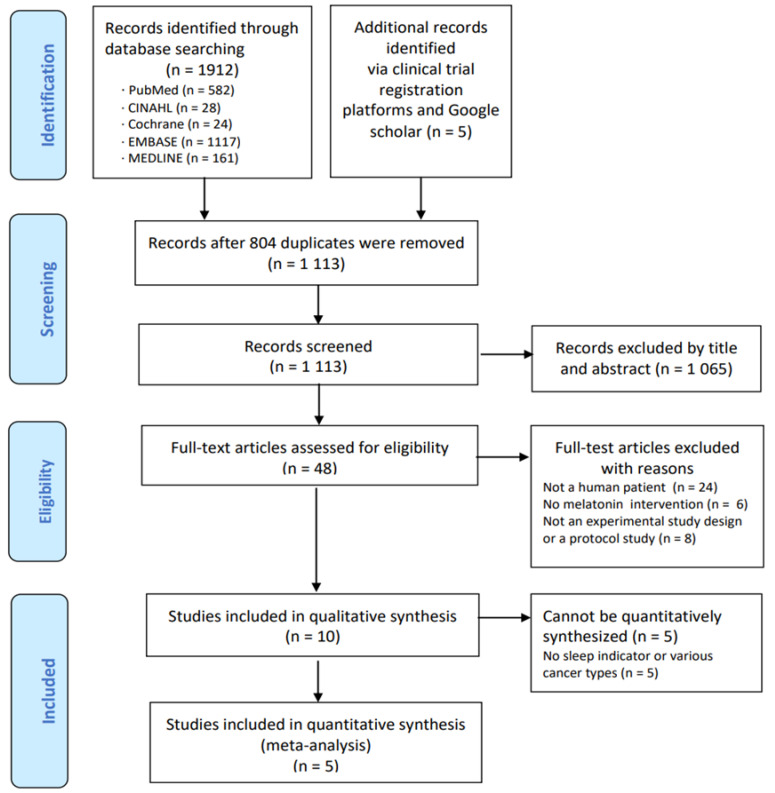
Flow diagram of study selection.

Overall, 1917 records were identified by database searches. After the removal of 804 duplicates, a further 1065 articles were excluded by title and abstract on the basis of the selection criteria. Subsequently, the full text of 48 articles were assessed. Among them, 38 irrelevant records were additionally excluded for the following reasons: not a human study, no melatonin intervention, or not an experimental study. Ten documents were analyzed for the context of melatonin intervention studies in breast cancer patients [19,20,21,22,23,24,25,26,27,28] with qualitative methods. Among these 10 melatonin intervention studies, several articles were excluded from the meta-analysis because they did not measure outcomes related to sleep or because their study population included various cancer types. Eventually, five studies were included in the meta-analysis [19,20,21,22,23] (Figure 1). In some studies, adverse effects were reported in a qualitative manner, but could not be evaluated by meta-analysis since most studies did not report adverse effects.

### 3.2. Context of the Studies Providing Melatonin to Breast Cancer Patients

Intervention studies have been conducted in various countries such as the United States, Japan, Brazil, and Denmark. Except for one, the selected studies were randomized clinical trials published within the last 10 years. Participants were diagnosed with various stages of breast cancer with treatments without primary sleep disorders and acute comorbidities. The number of the sample ranged from 32 to 95 across the studies. Although most participants were women in their 50s and 60s, some studies did not identify gender or included a man.

Oral melatonin supplements were provided for periods of 10 days to 4 months in the intervention group daily before bedtimes, whereas placebos or standard care were provided for the control groups. Melatonin was administered during chemotherapy, before and after surgery, or after completion of all treatments. A melatonin dose of less than 10 mg was administered at a time over a long period of intervention, and above 20 mg melatonin was administered in less than a month, a short intervention. Melatonin administered to the intervention group had doses ranging from 200 mg to 540 mg.

The tools used to measure sleep quality were the Pittsburg Sleep Quality Index and a 10-point visual analog scale. Sleep efficiency and sleep fragmentation were evaluated via actigraphy. Many studies have proven validation of the tools. The included studies reported that melatonin supplements improved sleep quality, sleep efficiency, and waking patterns (Table 1). Depending on the tool, the lower the score, the better the quality of sleep, or the higher the score, the better the sleep efficiency. It was confirmed by presenting standardized values for meta-analysis.

Besides sleep quality, other factors such as mood change, clinical response, pain, fatigue, and cognitive function were also assessed. Oral melatonin supplements reduced depressive symptoms, pain, fatigue, and side effects of chemotherapy. However, short-term melatonin treatment did not influence the estradiol and IGF level. Although some neuroprotective effects were reported, the effect on cognitive function could not be confirmed. Moreover, the melatonin administration group did not show more serious adverse effects than the control group except headaches and nightmares as minor adverse effects. There was also a study that reported decreased toxicity of cancer chemotherapy with melatonin administration (Table 1).

### 3.3. Risk of Bias Level

**Figure 2:**
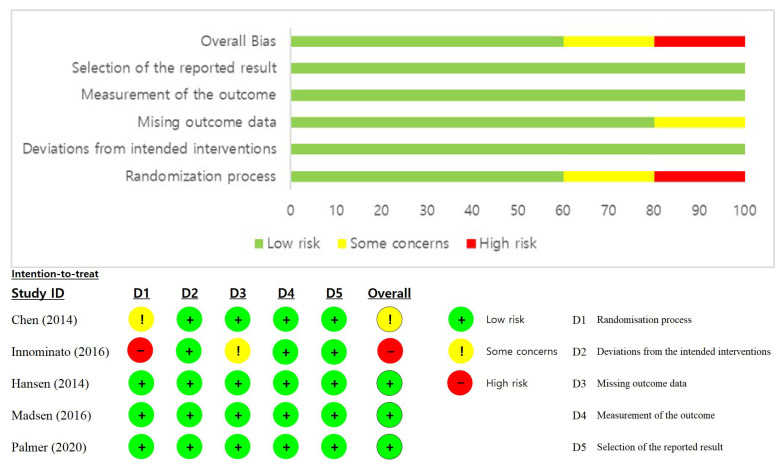
Risk of bias level for the included studies [19,20,21,22,23].

Studies were stratified as low risk-of-bias, some concerns, and high risk-of-bias for each of these criteria. The cumulative bias level for the included studies in the meta-analysis was evaluated as “low risk” in general, and two studies were assessed as “some concerns” or “High” in the randomization process (Figure 2).

### 3.4. Effect of Melatonin Supplementation on the Sleep of Breast Cancer Patients

**Figure 3:**
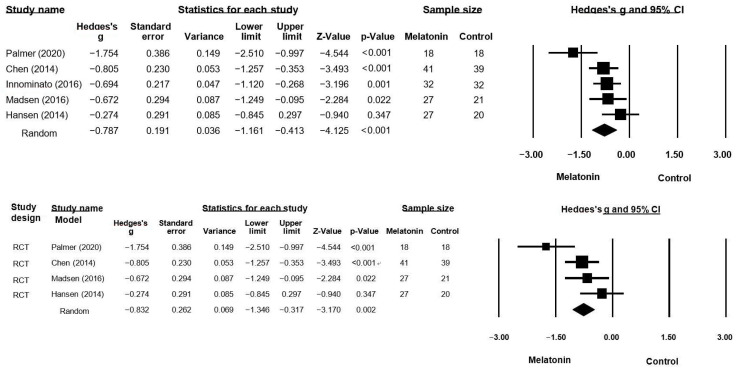
Forest plot of effects of melatonin on improvements in sleep quality [19,20,21,22,23].

Five studies included sleep-related variable as an outcome. The total number of participants included in the analysis was 243. The estimated average effect size (Hedges’ g) was −0.79 (*p* < 0.001) in a random-effects model, which was −0.79 SD of breast cancer patients who took melatonin supplements. This means that there was a difference of 7.9 points out of 10 points, thus indicating a moderate average effect size. The 95% confidence interval of the effect size was −1.16 to −0.41 (Figure 3). This result showed that when melatonin was provided, the sleep quality of breast cancer patients improved on average, and the effect might be very strong, moderate, or insignificant depending on the sample. Given that the confidence interval did not contain a zero, the null hypothesis that the effect size was zero (Z = −4.13) was dismissed. Thus, compared with placebo, melatonin supplementation in patients diagnosed with breast cancer had positive effects in terms of improved sleep quality.

The degree of heterogeneity in the analyzed studies was moderate (I^2^ = 58.45; Q = 9.63, df[Q] = 4, *p* = 0.047). Considering that the number of analyzed reports did not exceed 10, the degree of publication bias could not be determined by a funnel plot. For the sensitivity analysis via subgroup analysis of the study design was conducted. Except a single arm study, 4 RCT studies show the estimated average effect size (Hedges’ g) was −0.83 (*p* = 0.002) in a random-effects model (95% CI range was −1.35 to −0.32) (Figure 3), the degree of heterogeneity was similar (I^2^ = 68.51; Q = 9.53). When one more study with skewed results was excluded, three studies showed the estimated average effect size (Hedges’ g) was −0.62 (*p* < 0.001) in a random effects model (95% CI range was −0.93 to −0.31).

## 4. Discussion

The anticancer effect and underlying mechanism of melatonin have been reported previously [29]. However, there is a paucity of studies that analyzed the systematic effects of melatonin on sleep quality in cancer patients [15,30]. Against the background of disparate opinions on the effectiveness of melatonin for controlling sleep disorders, the current study evaluated the effectiveness of melatonin for treating sleep disturbances in breast cancer patients, with a focus on improving the quality of life of breast cancer patients.

Based on meta-analysis of the experimental studies, we confirmed that melatonin can improve the sleep quality of breast cancer patients. One literature was a single-group experimental design, not RCT, except that study, the estimated average effect size was increased. And the effect size decreased when one more study with skewed result was removed. But all were statistically significant. Even though melatonin per se could not improve sleep quality among orthopedic disease patients [31] and other cancers [14], melatonin could modulate circadian rhythms and act as an antioxidant in breast cancer patients [32]. In vitro experimental studies have shown that melatonin did not promote cancer cell death but inhibited their proliferation [33]. Similar results were demonstrated in rodent models [34]. Mechanistically, the antioxidant activity of melatonin is exerted via the p53–p21 pathway [35], and the circadian rhythm is modulated via tumor fat metabolism [36]. Consistent with these mechanisms and regardless of the study type, previous research has consistently shown an elevated risk of developing breast cancer among people working the night shift, which causes an imbalance in melatonin [37]. Nevertheless, more clinical experimental studies should be conducted in the future to increase the evidence for the effects of melatonin. The results of this study can be used to propose an effective intervention strategy when consulting breast cancer patients who are struggling with sleep problems. Melatonin could be recommended as a standard treatment or complementary therapy for the symptoms related to breast cancer treatments [10]. In addition, it should be a priority for healthcare providers to pay attention to sleep quality and related symptoms of breast cancer patients [5].

Most of the studies synthesized had women breast cancer participants in mid-50s with cancer treatments. Including sleep disturbance, menopausal symptoms have various symptoms such as depression, hot flash, and cognitive disfunctions. In addition to natural menopause, they have been experiencing artificial menopause caused by hormonal drug therapy. Furthermore, women with breast cancer had higher fatigue level and lower sleep quality than cancer free women in menopausal status [5]. Oncological Nursing Society proposed guidelines including pharmacological, behavioral and dietary strategies for hormonal cancer treatment related symptom [38]. Future studies should explore the effects of behavioral therapy or dietary therapy on sleep disturbance and be able to be presented in the form of guidelines like other symptoms.

The effect of melatonin on sleep quality differed from various cancer types [14]. In this study, we did not compare the effect of melatonin with other tumors to ascertain whether melatonin was specifically effective for sleep disturbances in breast cancer patients. The results of the current study indicate that further investigations into treating sleep problems and exploring mechanisms in hormone-related cancer patients are warranted. Additionally, future studies could compare the effects in other tumors, or on the improvement of symptoms such as pain, fatigue, cognitive function and depressive mood of breast cancer.

The melatonin administration group did not show more serious adverse effects than did the control group. A review paper on adverse effects of melatonin administration studies involving various types of disease also reported that there were either no adverse effects or that the adverse effects were minor and controllable [39]. Nevertheless, it is necessary to inform breast cancer patients about the possible occurrence of headaches and nightmares as minor adverse effects of oral melatonin supplementation [19].

Melatonin plays a protective role against chemotherapy side effects in patients with hormone-related cancers, such as breast, prostate, and ovarian cancers, in both in vivo and in vitro experiments [29]. Among the included studies, there was a study [23] that reported that the side effects of anticancer drugs were reduced when melatonin was administered along with chemotherapy. In future studies, a study can be proposed to confirm the evidence for the effect of melatonin supplement on the side effects of chemotherapy.

This study has some limitations. There were not many papers included in the meta-analysis enough to be divided into subgroups, and the incomplete addressing of confounding remains a limitation of the included studies. Although meta-analysis uses standardized effect size values, heterogeneity affects the interpretation of the results. The treatment regime used in the patients, cancer stage, differed slightly in the reports, so a random model was also used in meta-analysis. Additionally, the difference in adverse effects of melatonin supplementation could not be assessed.

## 5. Conclusions

This meta-analysis showed that melatonin intake improved the quality of sleep in breast cancer patients, especially for patients with adjuvant treatment. The effect of melatonin in improving sleep quality without serious adverse effects was reported; however, not all studies reported adverse effects. Hence, further studies are required. This finding also suggested that melatonin could be used as an adjunct therapy only to reduce in chronic and high-stress cancer patients. Given that only a few papers were analyzed in this study, additional meta-analyses are necessary when new research data are obtained.

For future direction, it is important for breast cancer patients to discuss the potential benefits and risks of melatonin with their healthcare provider before using it as a treatment or supplement. A healthcare provider can help to evaluate each individual’s unique situation and make personalized recommendations about the use of melatonin or other treatments.

## Figures and Tables

**Table 1:** Characteristics of the studies providing melatonin to breast cancer patients.

First Author (Year)	Sample (n)	Intervention	Comparison, Study Design	Primer Outcome	Measurement Tool	Adverse Events	Findings (MA)
Chen (2014) [19]	Mean 59 (38–81) years old, menopaused women, stage III breast cancer who had completed active cancer treatment (including hormonal therapy) (95)	MLT 3 mg, PO, 4 months	Placebo, RCT	Sleep Depression Hot flashes	PSQI, CES-D, NCCTG hot flash diary	Headache, fatigue, and bad dreams	MLT improved in subjective sleep quality (O).
Hansen, Madsen (2014) [20]	Mean 51- and 60-years old women, lumpectomy or mastectomy for breast cancer (54)	MLT 6 mg, PO, 3 months	Placebo, RCT	Sleep Postoperative cognitive dysfunction	Sleep diary and VAS, ISPOCD Test Battery	No report	MLT increased sleep efficiency and total sleep time (O).
Hansen, Anderson (2014) [24]	46~ 68 years old Women, lumpectomy or mastectomy for breast cancer	MLT 6 mg, PO, 3 months	Placebo, RCT	Depressive symptoms, Anxiety	MDI	No report	MLT reduced depressive symptoms. No reported sleep related outcomes (X).
Innominato (2016) [21]	Mean 55 years old (33–69), 1 male including. Metastatic breast cancer, receiving hormonal or trastuzumab therapy (32)	MLT 5 mg, PO, 2 months	Repeated measured design (before starting and last week of the MLT	Sleep efficiency, sleep fragmentation, Cortisol Expression of the core clock genes PER2 and BMAL1	r24 and pRA via actigraphy, Global quality of life, serum ELISA, PCR (no absolute values of the results)	No report	MLT improved sleep and quality of life (O).
Lissoni (1999) [25]	Metastatic solid tumor patients (including 77 breast cancer patients)	MLT 20 mg PO, 18 weeks	Standard care, RCT	Clinical response (survival rate, regression rate)	Radioimmunoassay	Reduced thrombocytopenia, neurotoxicity, cardiotoxicity, stomatitis, asthenia	No reported sleep-related outcomes (X).
Madsen (2016) [22]	Mean 51–59 years old women, lumpectomy or mastectomy for breast cancer (48)	MLT 6 mg, PO, 3 months	Placebo, RCT	Sleep quality, sleep efficacy	Sleep quality VAS, KSS, actigraphy	No differences in side effects were found.	MLT changed sleep efficiency and waking after sleep onset after surgery (O).
Palmer (2019) [26]	18–75 years old female, Adjuvant chemotherapy for breast cancer (36)	MLT 20 mg, PO, 10 d oncological care	Placebo, RCT	Numerical pain rating score Heat pain threshold Heat pain tolerance Neuroplasticity	CPM task, QST, Serum BDNF, TrkB, S100B-protein,	No report	MLT decreased pain and neural plasticity. No reported sleep related outcomes (X).
Palmer (2020) [23]	Mean 54 years old female, Adjuvant chemotherapy for breast cancer ACBC (36)	MLT 20 mg, PO, 10 days, daily, before and during the first cycle of ACBC	Placebo, RCT	Sleep Depressive symptoms Cognitive function Quality of life	PSQI, BDI-2, TMT A-B, RAVLT, COWAT, Go/No-Go task, EORTC QLQ-C30	Reduced side effects.	MLT counteract the adverse effects of ACBC on cognitive function, sleep quality, and depressive moods (O).
Pashaki (2021) [27]	Mean 46, 50 years old, Adjuvant chemotherapy and radiotherapy for breast cancer (37)	MLT 18 mg PO during adjuvant chemotherapy, 1 week before until 1 month after radiotherapy	Placebo, RCT	Fatigue	Brief Fatigue Inventory	No report	MLT decreased fatigue (X).
Schernhammer (2012) [28]	Mean 60 years old women, stage 0–III breast cancer who had completed active cancer treatment (including hormonal therapy) (48)	MLT 3 mg, PO, 4 months, daily	Placebo, RCT	Estradiol IGF-1 IGFBP-3	Blood test (ELISA)	Headache, fatigue, and bad dreams	No reported sleep related outcomes (X).

ACBC: Adjuvant chemotherapy for breast cancer; ASA: American Society of Anesthesiologists classes; BDI-2: Beck Depression Inventory; CES-D: Center for Epidemiologic Studies-Depression; COWAT: Controlled Oral Word Association Test; CPM: Conditioned Pain Modulating; EORTC QLQ-C30: The EORCT Quality of Life Questionnaires; ELISA: enzyme-linked immunosorbent assay; IGF-1: insulin-like growth factor I; IGFBP-3: insulin-like growth factor–binding protein 3; KSS: The Karolinska Sleepiness Scale; IL: interleukin; ISPOCD: International Study of Post-Operative Cognitive Dysfunction; MA: Meta-analysis; MDI: Major Depression Inventory; MLT: Melatonin; NCCTG: North Central Cancer Treatment Group; PO: per os; POCD: Postoperative Cognitive Dysfunction; PSQI: Pittsburgh Sleep Quality Index; QST: Quantitative Sensory Testing; RAVLT: Rey Auditory-Verbal Learning Test; RCT: Randomized Clinical Trial; TMT: Trail Making-Test; TMX: tamoxifen; VAS: Visual Analog Scale.

## Data Availability

All relevant data are within the manuscript.
